# Refractory *Staphylococcus hominis* Endophthalmitis With Corneal Involvement Following Cataract Surgery: A Case Report

**DOI:** 10.1155/crop/7782331

**Published:** 2026-07-06

**Authors:** Issac Levy, Aaminah Haq, Mayank A. Nanavaty

**Affiliations:** ^1^ Sussex Eye Hospital, University Hospitals Sussex NHS Foundation Trust, Brighton, UK; ^2^ Ophthalmology Department, Rabin Medical Center, Petach Tikva, Israel, clalit.co.il; ^3^ Faculty of Medicine, Tel Aviv University, Tel Aviv, Israel, tau.ac.il; ^4^ Brighton and Sussex Medical School, University of Sussex, Brighton, UK, sussex.ac.uk

**Keywords:** biofilm, cataract surgery, corneal infiltrate, endophthalmitis, *Staphylococcus hominis*, tectonic keratoplasty

## Abstract

We report an unusual case of delayed‐onset, refractory endophthalmitis caused by *Staphylococcus hominis* following uncomplicated cataract surgery, characterized by recurrent anterior chamber hypopyon and progressive corneal infiltration requiring tectonic keratoplasty for definitive management. A 74‐year‐old patient with well‐controlled Type 2 diabetes mellitus underwent uncomplicated phacoemulsification with intraocular lens implantation, achieving best corrected visual acuity of 6/6 (Snellen) at 1 month postoperatively. The patient subsequently developed acute vision loss with clinical endophthalmitis. Despite multiple interventions, including vitreous tap, two pars plana vitrectomies with intravitreal antibiotics, and intensive topical therapy, the infection persisted for 5 months with recurrent corneal wound infiltration and hypopyon. All initial vitreous and aqueous cultures remained negative. Following referral to our tertiary center, sequential anterior chamber washes, corneal biopsies, and intracameral antibiotics were performed. *S. hominis* was eventually isolated from corneal sampling. After the identification of *S. hominis*, treatment was converted to systemic linezolid and intensive topical vancomycin. Given persistent corneal involvement despite maximal medical therapy, a superior 6‐mm tectonic keratoplasty was performed. Following this definitive surgical intervention combined with targeted antimicrobial therapy, the infection was successfully controlled, and intraocular inflammation was completely resolved. This represents the first reported case of *S. hominis* endophthalmitis presenting with recurrent anterior chamber hypopyon and progressive corneal wound involvement successfully treated with tectonic keratoplasty. The case highlights that *S. hominis* should be considered in the setting of persistent postoperative endophthalmitis, particularly in diabetic patients, and that tectonic keratoplasty may be required for definitive source control when medical therapy fails to eradicate the infection.

## 1. Introduction

Postoperative endophthalmitis remains one of the most devastating complications of cataract surgery. Contemporary studies report an incidence ranging from 0.012% to 0.102%, representing a significant decline over recent decades due to improved surgical techniques, routine povidone–iodine antisepsis, and prophylactic intracameral antibiotics [1]. The most prevalent causative organisms are Gram‐positive bacteria, predominantly coagulase‐negative staphylococci, with *Staphylococcus epidermidis* as the most common species [2].

*Staphylococcus hominis*, another coagulase‐negative *Staphylococcus*, is rarely associated with postoperative endophthalmitis, with only a handful of cases reported in the ophthalmic literature [3]. As a commensal organism of human skin, *S. hominis* is generally considered to have low virulence [4]. However, this organism possesses the ability to produce biofilm, particularly in patients with diabetes mellitus or other immunocompromising conditions, which may facilitate antibiotic resistance and chronic infection [3, 5].

We present a rare case of delayed‐onset, chronic endophthalmitis caused by *S. hominis* following routine cataract surgery, notable for its refractory nature, persistent corneal involvement, and ultimate requirement for tectonic keratoplasty to achieve infection control.

## 2. Case Report

A 74‐year‐old male with a medical history of well‐controlled Type 2 diabetes mellitus (HbA1c 6.8%) and prior uneventful right eye cataract surgery underwent uncomplicated left eye phacoemulsification with intraocular lens implantation at a private medical center. The surgery was performed under topical anesthesia with a 2.8‐mm clear corneal temporal incision. Postoperatively, the patient received topical dexamethasone 0.1% and chloramphenicol 0.5% four times daily. At 1 month postoperatively, his best corrected visual acuity (BCVA) was 6/6 (Snellen chart) with a quiet anterior chamber.

Approximately 6 weeks postoperatively, the patient developed acute vision loss to light perception in the left eye associated with pain, photophobia, and conjunctival injection. Clinical examination revealed features consistent with endophthalmitis, including anterior chamber inflammation with a 2‐mm hypopyon, dense vitritis, and an absent fundal view. He was treated emergently with a vitreous tap yielding 0.3 mL of sample, followed by intravitreal injection of ceftazidime 2.25 mg and vancomycin 1 mg. Fortified topical antibiotics (cefuroxime 5% and gentamicin 1.5%) were commenced hourly, along with cyclopentolate 1% three times daily and oral ciprofloxacin 750 mg twice daily. The empirical antimicrobial regimen, comprising intravitreal ceftazidime and vancomycin as first‐line agents with adjunctive fortified topical antibiotics and oral ciprofloxacin, was consistent with current evidence‐based guidelines for acute postoperative endophthalmitis and reflected the clinical urgency of a rapidly deteriorating, sight‐threatening presentation.

Despite initial treatment, the patient′s condition deteriorated over 48 h with worsening vitritis and persistent hypopyon. He underwent pars plana vitrectomy (PPV) with repeat intravitreal antibiotics (ceftazidime 2.25 mg and vancomycin 1 mg) and vitreous biopsy. All vitreous and aqueous samples remained culture‐negative. Vision transiently recovered to 6/6 by 2 months with resolution of inflammation but rapidly declined to hand movements within 1 week after discontinuing topical antibiotics, accompanied by the recurrence of hypopyon.

A second PPV with vitreous biopsy and repeat intravitreal antibiotics (amikacin 0.4 mg and vancomycin 1 mg) was performed; however, all cultures and polymerase chain reaction (PCR) testing remained negative for bacteria and fungi. Due to a lack of improvement and progression despite broad‐spectrum antibiotics, the patient was referred to a tertiary center where B‐scan ultrasonography revealed a quiet vitreous cavity but a dense corneal infiltrate at the superior main surgical wound. A corneal suture was removed for culture, and intensive topical and systemic antibacterial therapy was commenced. The culture was reported as negative. When the patient showed continued progression with increased hypopyon despite this regimen, empirical antifungal therapy (oral fluconazole 400 mg daily and topical amphotericin B 0.15% hourly) was added to intravitreal amikacin 0.4 mg and vancomycin 1 mg. The patient showed transient improvement with partial resolution of hypopyon and visual recovery to 6/36, despite repeatedly negative bacterial and fungal cultures and PCR results.

**Figure 1:**
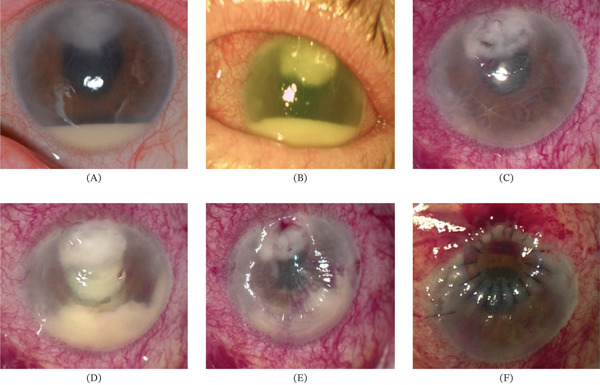
Sequential anterior segment photographs demonstrating disease progression and treatment response. (A) Initial presentation showing superior corneal wound infiltrate with surrounding stromal edema and anterior chamber hypopyon. (B) Three days following the first anterior chamber washout with intracameral antibiotics, showing partial improvement. (C) End of second anterior chamber washout demonstrating persistent corneal infiltration. (D) Three days postsecond anterior chamber washout with recurrent hypopyon. (E) Postcorneal biopsy showing a full‐thickness defect at the biopsy site. (F) Posttectonic corneal graft showing a clear graft with well‐opposed wound edges and resolved inflammation.

Five months after the initial presentation with endophthalmitis, recurrent episodes of corneal wound infiltration and hypopyon led to referral to our tertiary center (Sussex Eye Hospital). Clinical examination revealed a superior corneal wound infiltrate measuring 3 × 2 mm with surrounding stromal edema, 1.5 mm hypopyon, and moderate anterior chamber reaction (Grade 3+). Seidel testing was performed and was negative, with no evidence of wound leak. The posterior segment showed vitreous haze (Grade 2+) but no evidence of retinitis or vitritis (Figure 1).

We performed sequential anterior chamber washouts with aqueous sampling on two separate occasions, combined with intracameral injections of vancomycin 1 mg/0.1 mL, amikacin 0.4 mg/0.1 mL, and voriconazole 100 *μ*g/0.1 mL. The intraocular lens was examined intraoperatively in detail, including the capsular bag and lens–capsule–zonular complex. No suspicious material, deposits, or visible biofilm‐like plaque were identified. Intensive fortified topical therapy was instituted, including cefuroxime 5% and gentamicin 1.5% hourly, along with antifungal therapy (voriconazole 1% and natamycin 5%) every 2 h. Corneal scraping was performed from the wound infiltrate for microbiology testing (Figure 1). Samples were collected using a glass slide, chocolate agar, blood agar, and Sabouraud′s agar, together with *Σ*‐VIROCULT viral swab (Sigma‐Virocult, MWE, United Kingdom), TRANSWAB Amies Charcoal bacterial swab (MWE, United Kingdom), and *Σ*‐TRANSWAB (Sigma‐Transwab, MWE, United Kingdom) bacterial and viral swab transport media. The microbiology laboratory performed direct microscopy, culture, and sensitivity testing on the submitted ocular specimens. Specimens were inoculated into both aerobic and anaerobic incubation bottles and examined by Gram staining. In addition, multiplex reverse‐transcription PCR was performed for Herpes Simplex Virus Type 1, Herpes Simplex Virus Type 2, varicella‐zoster virus, adenovirus, *Chlamydia trachomatis*, *Neisseria gonorrhoeae*, and *Treponema pallidum*. Bacterial 16S and fungal 18S ribosomal RNA PCR, *Mycobacterium* DNA testing, and *Acanthamoeba* DNA testing were also undertaken.

**Figure 2:**
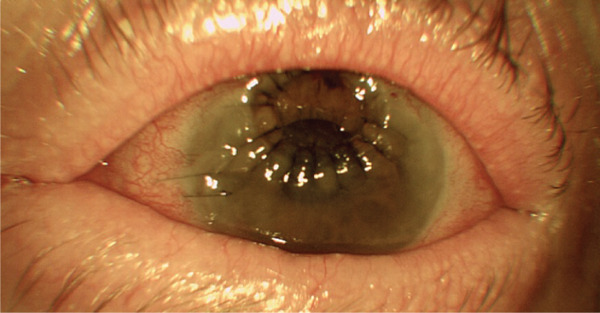
Clinical appearance 4 days following tectonic corneal graft transplantation. The image demonstrates clear donor corneal tissue with interrupted 10‐0 nylon sutures in place, absence of infiltration, clear anterior chamber without hypopyon, and well‐centered intraocular lens. Note the superior graft–host junction is well‐apposed without signs of infection or rejection.

Given persistent corneal involvement despite multiple interventions, partial‐thickness corneal biopsy (lamellar dissection to mid‐stromal depth) was performed from the infiltrated superior wound site, followed 1 week later by full‐thickness corneal biopsy when infection persisted. Tissue specimens were sent for another bacterial culture; fungal culture, as above; and a histopathological examination. After multiple negative investigations, *S. hominis* was finally isolated from the first corneal biopsy cultures, which was later approved by the histology report of the full‐thickness biopsy. Antibiotic susceptibility testing revealed sensitivity to vancomycin (minimum inhibitory concentration 1 *μ*g/mL), linezolid, and gentamicin, with resistance to penicillin and erythromycin. On microbiological advice, treatment was converted to intensive topical vancomycin 50 mg/mL every hour and systemic linezolid 600 mg twice daily. Figure 2 shows the picture of the eye 4 days after the tectonic graft.

Despite 48 h of targeted antimicrobial therapy with clinical microbiological guidance, the corneal wound infiltrate persisted with ongoing anterior chamber inflammation. Given the refractory nature of the infection with persistent wound‐related corneal involvement despite maximal medical and intracameral therapy, the decision was made to proceed with surgical source control. The patient underwent a superior 6‐mm tectonic penetrating keratoplasty to excise all infected tissue (Figure 1). The surgical procedure involved excision of the full‐thickness corneal tissue containing the infiltrated wound, meticulous anterior chamber irrigation with balanced salt solution containing vancomycin (20 *μ*g/mL), removal of inflammatory membranes, and a 6‐mm donor corneal graft secured with 16 interrupted 10‐0 nylon sutures. The excised corneal tissue was sent for microbiological culture and histopathological examination, which confirmed the presence of *S. hominis* and demonstrated acute and chronic inflammatory cell infiltration.

Postoperatively, topical vancomycin 50 mg/mL was continued hourly for 2 weeks and then gradually tapered, whereas systemic linezolid 600 mg twice daily was continued for a total of 6 weeks. Topical dexamethasone 0.1% was introduced at low frequency (twice daily) 2 weeks postoperatively after confirming infection control and gradually increased to manage graft‐related inflammation. Following the definitive tectonic keratoplasty combined with targeted antimicrobial therapy, the infection was successfully controlled with complete resolution of intraocular inflammation. Figure 2 shows the picture of the eye 4 days after tectonic keratoplasty. At 7 months postoperatively after his cataract surgery, the patient maintained a clear corneal graft with counting fingers vision, irregular astigmatism from the graft, and haziness of his corneal rim. Intraocular pressure remained normal (14 mmHg), and there was no evidence of recurrent infection or graft rejection. At the time of submission, the patient has not yet undergone optical penetrating keratoplasty. He remains under regular follow‐up and is awaiting optical keratoplasty to restore full visual function, with the tectonic graft remaining clear and stable with no signs of rejection or recurrent infection.

## 3. Discussion

*S. hominis* is an infrequent cause of postoperative endophthalmitis, with only sporadic cases reported in the ophthalmic literature [3]. Although *S. hominis* is part of the normal skin flora and generally considered to have low pathogenic potential, it can cause severe ocular infections under specific circumstances, particularly in immunocompromised hosts or those with diabetes mellitus [3]. The biofilm‐forming capacity of *S. hominis*, especially in diabetic or immunosuppressed patients, may contribute to antibiotic resistance and persistent infection despite appropriate antimicrobial therapy [5].

The presentation in our case was particularly unusual in several aspects. The patient initially achieved an excellent postoperative outcome before developing endophthalmitis approximately 6 weeks after surgery, consistent with a delayed‐onset, low‐grade infection rather than acute perioperative contamination. This temporal pattern suggests a chronic process, potentially related to organisms sequestered within avascular tissue or protected within a biofilm, allowing persistence with intermittent anterior chamber spillover and recurrent intraocular inflammation.

Despite extensive management, including multiple vitrectomies, anterior chamber washouts, and repeated intravitreal and intracameral antibiotic administration, the infection persisted with a distinctive pattern of recurrent hypopyon—a presentation not previously described in the *S. hominis* endophthalmitis literature [3]. A key feature of this case was the persistent corneal wound infiltrate, implicating the surgical incision as the likely reservoir of infection. Although corneal involvement was not prominent initially, the repeated localization of inflammation to the wound, subsequent isolation of *S. hominis* from corneal tissue, and definitive resolution following tectonic keratoplasty strongly support this hypothesis. While an occult intraocular source cannot be entirely excluded, the consistently negative vitreous and aqueous cultures, together with later microbiological confirmation from corneal tissue, make the corneal wound the most plausible origin. Although the initial presentation was consistent with acute endophthalmitis without overt corneal signs, the subsequent clinical course—with recurrent wound infiltration, persistently negative vitreous cultures, and eventual microbiological confirmation from corneal tissue—retrospectively supports a primary corneal wound infection with secondary anterior chamber involvement. This diagnostic reclassification only became apparent in retrospect once a corneal source was identified.

This corneal localization of *S. hominis* endophthalmitis has not been reported previously and likely contributed to the infection′s refractory nature. The organism′s ability to form biofilm, particularly in the setting of diabetes mellitus, may have facilitated its persistence at the avascular corneal wound site despite systemic and topical antibiotic therapy [5]. Biofilm‐associated bacteria exhibit significantly reduced susceptibility to antimicrobial agents, often requiring concentrations 10–1000 times higher than those required to eradicate planktonic bacteria [6]. Several factors complicated the diagnostic workup in this case. Initial vitreous and aqueous cultures remained persistently negative despite clinical evidence of ongoing inflammation. This highlights the limitations of conventional vitreous sampling, which yields a positive culture in only 36%–69% of clinically diagnosed cases of endophthalmitis [2]. The low microbial load in chronic infections, fastidious organism growth requirements, or sequestration of organisms within biofilm or avascular tissue may all contribute to culture‐negative results. In our case, the diagnosis was ultimately established only through a corneal tissue biopsy, which provided sufficient material for both culture and histopathological examination.

The empirical antifungal treatment initiated at the referring center, when the patient deteriorated despite antibacterial therapy, warrants consideration. While the infection ultimately proved to be bacterial, the clinical deterioration despite broad‐spectrum antibiotics and persistent negative cultures justified empirical antifungal coverage, as fungal endophthalmitis remains an important differential diagnosis in refractory cases [7]. The transient improvement observed during this period may have been coincidental or attributable to concurrent intensive antibacterial therapy and surgical interventions rather than to the antifungal agents. We acknowledge that dual antifungal therapy was an aggressive empirical step; however, it was justified given persistent deterioration despite maximal antibacterial therapy and repeated culture‐negative investigations. Fungal endophthalmitis remains a critical differential in refractory culture‐negative cases and warrants empirical cover when all other interventions have failed.

The role of therapeutic or tectonic keratoplasty in managing severe infectious keratitis, including cases complicated by endophthalmitis, is well established as a potentially globe‐saving procedure when intensive medical therapy fails to control infection or maintain corneal integrity [8]. Tectonic keratoplasty serves multiple purposes, including removal of infected tissue to reduce microbial load and biofilm burden, restoration of corneal integrity, prevention of further anterior chamber contamination, and provision of tissue for definitive microbiological and histopathological diagnosis [8]. In cases of endophthalmitis with associated corneal involvement, corneal transplantation may be necessary to achieve source control when the corneal wound harbors organisms protected within biofilm or avascular tissue inaccessible to antibiotics.

In our case, the decision to proceed with tectonic keratoplasty was made after multiple medical and surgical interventions failed to control the infection. The successful outcome following keratoplasty strongly supports the hypothesis that the corneal wound site served as the primary reservoir for persistent infection. The 6‐mm graft diameter was selected to ensure complete excision of infected tissue while minimizing graft size, as smaller diameter grafts are associated with reduced rates of corneal graft failure and improved long‐term survival [9].

The management of postoperative inflammation following tectonic keratoplasty for infectious indications requires careful balance between controlling inflammation and avoiding corticosteroid‐associated complications, including infection reactivation, elevated intraocular pressure, and impaired wound healing. In our case, low‐dose topical corticosteroids were introduced only after confirming infection control at 2 weeks postoperatively, with gradual escalation to manage graft‐related inflammation while maintaining vigilant monitoring for signs of recurrent infection.

This case illustrates several important clinical lessons. First, *S. hominis* should be considered a potential pathogen in postoperative endophthalmitis, particularly in diabetic or immunocompromised patients who may be predisposed to biofilm‐forming organisms [3, 5]. Second, persistent or recurrent anterior segment inflammation with corneal wound involvement despite appropriate intravitreal, intracameral, topical, and systemic antimicrobial therapy should prompt consideration of the surgical wound as a potential reservoir of infection [10]. Third, a corneal tissue biopsy may be necessary to establish a microbiological diagnosis when conventional sampling methods yield negative results in clinically evident infection [11]. Fourth, tectonic keratoplasty should be considered a definitive treatment option to achieve source control and eradicate infection when medical therapy fails in cases of endophthalmitis with persistent corneal involvement [8].

The limitations of this case report include the lack of a systematic evaluation of biofilm production by the isolated *S. hominis* strain, which would have provided additional insight into the mechanisms underlying antibiotic resistance and chronic infection. Additionally, anterior segment optical coherence tomography was not performed to characterize the depth and extent of corneal infiltration before surgery, which could have aided surgical planning. Finally, confocal microscopy of the corneal infiltrate was not available, which might have provided earlier identification of bacterial colonization.

## 4. Conclusion

To our knowledge, this represents the first reported case of *S. hominis* endophthalmitis presenting with refractory corneal wound involvement and recurrent anterior chamber hypopyon successfully managed with tectonic keratoplasty. This case demonstrates that *S. hominis*, despite its reputation as a low‐virulence commensal organism, can cause severe, refractory endophthalmitis, particularly in diabetic patients with enhanced biofilm‐forming capacity. When endophthalmitis is associated with persistent corneal wound involvement refractory to comprehensive medical management, including intravitreal, intracameral, topical, and systemic antibiotics, tectonic keratoplasty should be considered a definitive treatment to achieve surgical source control and eradicate sequestered infection. Early recognition of corneal involvement as a potential reservoir for persistent infection and timely surgical intervention may be critical for achieving favorable outcomes in refractory postoperative endophthalmitis.

## Funding

No funding was received for this manuscript.

## Consent

No written consent has been obtained from the patients, as there is no patient‐identifiable data included in this case report/series.

## Conflicts of Interest

The authors declare no conflicts of interest.

## Patient Perspective

After routine cataract surgery, I initially enjoyed clear vision before suddenly losing sight in my left eye due to relentless infection, repeated operations, and intense treatment over many months, which eventually stabilized after a corneal graft, though my vision remains limited and I am still facing the possibility of further surgery.

## Data Availability

The data that support the findings of this study are available upon request from the corresponding author. The data are not publicly available due to privacy or ethical restrictions.
